# Ability paradox of cascading model based on betweenness

**DOI:** 10.1038/srep13939

**Published:** 2015-09-10

**Authors:** Jianwei Wang, Bo Xu, Yuedan Wu

**Affiliations:** 1 School of Business Administration, Northeastern University, Shenyang 110819, P. R. China

## Abstract

Must Investing more resources to protect every node in a network improve the robustness of the whole network subject to target attacks? To answer this question, we investigate the cascading dynamics in some typical networks. In real networks, the load on a node is generally correlated with the betweenness. Considering the weight of a node, we give a new method to define the initial load on a node by the revised betweenness. Then we present a simple cascading model. We investigate the cascading dynamics by disabling a single key node with the highest load. We find that in BA scale-free networks, the bigger the capacity of every node, the stronger the robustness of the whole network. However, in WS networks and some random networks, when we increase the capacity of every node, instead, the robustness of the whole network is weaker. In US power grid and the China power grid, we also observe this counterintuitive phenomenon. We give a reasonable explanation by a simple illusion. By the analysis, we think that resurrections of some nodes in a ring network structure after removing a node may be the reason of this phenomenon.

Over the past several years, the study on the network robustness^1^,^2^,^3^,^4^,^5^,^6^,^7^,^8^,^9^,^10^,^11^ has been attracted so much attention. In particular, many researchers focus on the vulnerability of natural and man-made complex systems under cascading failures induced by removing some critical nodes or edges. Cascading failures are ubiquitous in power grid, traffic networks, and computer networks^12^,^13^,^14^,^15^. In these networks, there exist the loads in forms of electricity, traffic flows, or data flows. Under normal circumstances, no cascading failure occurs and the system maintains its normal and efficient functioning, while the failures of some key nodes or edges may cause large amount of loads to redistribute among other nodes in the networks, which may trigger more nodes’ failure and even entire collapse of the network. Some typical real-world examples of cascading failures are the large-scale blackouts in some countries, e.g., the blackouts of America in 2003, Italy in 2003, London in 2003, and northern India in 2012. In addition, the Internet collapse caused by the submarine earthquake near Taiwan in December 2006 and frequent traffic paralysis in some cities are also caused by long and intricate cascades of events.

Considering the vital importance of the safety of infrastructure networks, many researchers investigate the cascading phenomenon from different aspects, and many valuable conclusions have been reached, focusing on a variety of modeling approaches of cascading failures^16^,^17^,^18^,^19^,^20^,^21^,^22^,^23^,^24^, the cascade mechanism and control measures^25^,^26^,^27^,^28^,^29^,^30^,^31^,^32^,^33^,^34^,^35^,^36^, effective protection and attack strategies^37^,^38^,^39^,^40^, cascading modeling in interdependent networks^41^,^42^,^43^,^44^,^45^,^46^,^47^,^48^,^49^,^50^,^51^,^52^,^53^, cascading modeling in infrastructure networks^54^,^55^,^56^,^57^,^58^,^59^,^60^,^61^,^62^,^63^, and so on. One of the key contents in previous works on cascading failures is how to assign the initial load on a node or an edge. In earlier studies, the initial load on a node or an edge was generally estimated by the global betweenness, of which the pioneering work by Motter *et al.*^64^ discuss cascade-based attacks on complex networks and demonstrate that the attack on a single important node (one of those with high load) may trigger a cascade of overload failures capable of disabling the network almost entirely. Hereafter, to better control and defense cascading failures in complex networks, based on the betweenness method, Motter^25^ introduce and investigate a costless strategy of defense based on a selective further removal of nodes and edges, right after the initial attack or failure, and find that this intentional removal of network elements can drastically reduce the size of the cascade. Crucitti *et al.*^65^ propose a simple model for cascading failures based on the dynamical redistribution of the flow on the network and also show that the breakdown of a single node is sufficient to affect the efficiency of a network up to the collapse of the entire system if the node is among the ones with the largest load. Although the betweenness method to assign the initial load on a node or an edge can better reflect the flow of physical quantities in many realistic situations, it may not be practical for very large networks in some real networks such as the Internet or WWW, owing to its consideration of the whole networks topological information. Therefore, taking into account the simplicity of the strategy with the local degree, based on the information of the degrees of two nodes connected by an edge, W. X. Wang and G. R. Chen^20^ propose a new approach to define the initial load on an edge and present a cascading model. Motivated by their works, many researchers define the initial load on a node or an edge from the perspective of the degree and construct different cascading models. Since the definition of the initial load in the degree method ignores the flow characteristic of the load transported between two nodes, the constructed cascading models are valid in many actual applications.

To this end, applying the information of the degree and considering the calculation of the betweenness of a node, we introduce a new method to assign the initial load on this node and construct a cascading model with a tunable parameter. We focus on how the breakdown of a single node with the highest load due to attacks or failures is sufficient to collapse the entire network. In some man-made networks and infrastructure networks, because of the dynamics of redistribution of flows on the network, we observe that all these networks undergo a global cascade of overload failures when highly loaded nodes are removed. However, in WS networks, ring-coupled networks, some ER networks, and Power grid, we surprisingly find a counterintuitive phenomenon, i.e., the improvement of the capacity of every node does not reduce the size of the cascade, instead, the robustness of the network is more weaker. While in BA networks and US air port networks, naturally, the increase of the capacity of every node reduces the damage of the cascade. We observe that the phenomenon of the ability paradox of the cascading propagation is ubiquitous in many networks. In a ring-coupled network with 13 nodes and 26 edges, we also find this phenomenon. By carefully analyzing the dynamic mechanism of the cascading propagation and the calculating process of the betweenness of a node, we speculate that a kind of the ring structure in the remaining network after removing a node may be a cause of the ability paradox. Starting from a coupled-ring network and according to the rewiring probability on each existing edge, we study the cascading propagation in a serial of networks with the ring structure and observe the ability paradox in our cascading model. Our work may have practical value for controlling various cascading-failure-induced disasters in the real world.

## The Model

In many infrastructure networks, the loads of different forms are sent among nodes, e.g., data packets in computer networks, traffic flows in traffic networks, and electric current in power grid. In general, when the load is sent from one node to another, it is efficient to take a road along the shortest paths connecting these two nodes. In previous works, there are many ways to measure the load on a node or an edge, but, considering actual applications, many researchers define the initial load on a node or an edge to the total number of shortest paths passing through this node or this edge.

**Figure 1:**
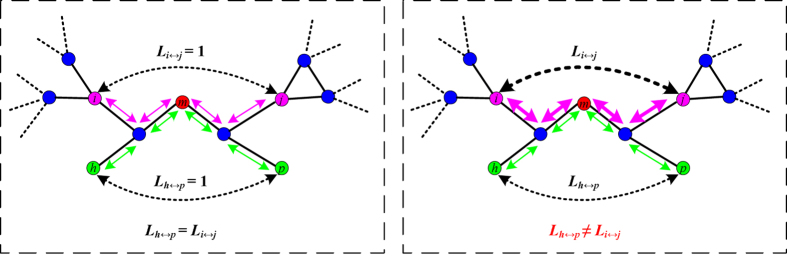
The scheme illustrates the correlation between the load transported between two nodes and the degrees of two ends. We use *L*_*i*↔*j*_ (*L*_*h*↔*p*_) denote the load transported between nodes *i* (*h*) and *j* (*p*). In traffic networks, the power grid, and the Internet, the bigger the degree of a node, the higher the load generated from it. Therefore, in general, 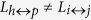.

Although the method by shortest paths is suitable for actual applications, it ignores the differences among nodes with the different degree (see Fig. 1). In the left sub-figure of Fig. 1, in the betweenness method, the load *L*_*i*↔*j*_ transported between nodes *i* and *j* is 1, i.e., only one new generated packet transmitted along the shortest paths connecting nodes *i* and *j*. Similarly, *L*_*h*↔*p*_ = 1. Thus, *L*_*h*↔*p*_ = *L*_*i*↔*j*_. However, in real networks, the bigger the degree of a node, the higher the load generated from it. In the right sub-figure of Fig. 1, owing to the effect of the differences of the node degree, 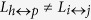, and generally 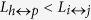. Therefore, according to the node degree and the shortest paths, we propose a new method to assign the initial load on a node, which can more suitable for defining the physical quantity of the load than previous methods.

Next, we simply introduce a new way to measure the initial load. First, we define the weights of nodes *i* and *j* to 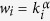 and 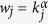, respectively. In general, the bigger the weight of a node, the higher the load generated from it. For simplicity, we assume the loads transmitted between nodes *i* and *j* to *L*_*i*↔*j*_ = *w*_*i*_*w*_*j*_, i.e., 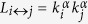. These loads are transmitted along the shortest paths connecting nodes *i* and *j*. If there is more than one shortest path connecting two given nodes, the packet is divided evenly at each branching point. We define 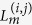 to denote the contribution of a packet transmitted between nodes *i* and *j* to the load on node *m*. Thus, the contribution of the load *L*_*i*↔*j*_ transmitted between nodes *i* and *j* to the load on node *m* is 
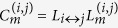
. The load *L*_*m*_ on node *m* is then


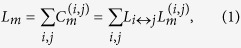


where the sum is over all pairs of nodes in a network.

When *α* = 0, our method to define the load on a node reduces to the one described in Refs 25,64,65, i.e., the load at a node is the total number of shortest paths passing through the node. The load *L*_*m*_(*t*) on node *m* at time *t* is the total number of all contributions of every ordered pairs of all nodes in the network at time *t*. Each node *m* is assigned to have a finite capacity *C*_*m*_, i.e., the maximum load that node can handle. Following Refs 19, 20, 21,29, 30, 31,39,40, we assume the capacity *C*_*m*_ of node *m* to be proportional to its initial load *L*_*m*_(0):


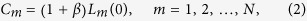


where the parameter *β* ≥ 0 is the tolerance parameter and *N* is the initial number of nodes in the network. The node *m* maintains its normal and efficient functioning if *L*_*m*_ ≤ *C*_*m*_; otherwise it fails and is removed from the network. In the initial stage at time *t* = 0, the network operates in a free-flow state. However, the removals of nodes due to attacks or failures, in general, change the distribution of the load on every node. Once the loads on some nodes exceed their capacities, these nodes will be removed from the remaining network, which will lead to a new redistribution of loads and, as a result, subsequent failures can occur. The cascading propagation will stop when the loads on all remaining nodes do not exceed their capacities.

In this paper, we only focus on the cascading propagation triggered by removing a node with the highest load. Here, we use *ϖ*_*t*_ to denote the resulting network after failed nodes are removed at time *t*. In a connected network *ϖ*_0_ at time *t* = 0, no cascading failures occur because 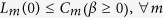. We assume that an initial attack is performed at time *t* = 1, i.e., one node with the highest load is removed from the network *ϖ*_0_ and the resulting network is denoted by *ϖ*_1_. Since the removal of a node may change the shortest paths between some node pairs and consequently a global redistribution of the load among the remaining nodes in the network *ϖ*_1_, we recalculate the load *L*_*i*_(1) on each remaining node, where 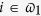. The updated load on some nodes may exceed their capacities, all the overloaded nodes then are removed from the *ϖ*_1_ and the resulting network is denoted by *ϖ*_2_. This leads to a new redistribution of loads and subsequent overloads may occur. The overloaded nodes are removed and the resulting network is denoted by *ϖ*_3_, and so on. When at time *ε*, the updated load satisfies *L*_*k*_(*ε*) ≤ *C*_*k*_ for all the nodes *k* in the remaining *ϖ*_1_, the cascading failures stop. The damage caused by a cascade is quantified in terms of the largest connected component *G* and the avalanche size *S*_*attack*_.

### The analysis of the cascading model

Two fundamental questions are, how the parameter *α* affect the network robustness against cascading failure and, the bigger the parameter *β*, the stronger the robustness of the network? To answer these questions, we focus on global cascades triggered by removing one node with the highest load. The reason that we choose the node with the highest load to the attacked object is because this node plays the vital role in the cascading failures in most previous studies. We illustrate how our model works in practice by considering two artificially created network topologies: a scale-free network (Barabasi-Albert)^66^ and a small-world network (Watts-Strogatz)^67^. The Watts-Strogatz small-world network (WS) starts as a lattice ring with *N* nodes, of which each node is connected with its 2*m* neighbors (*m* for each side). For each link, there is then a rewiring probability *p*. When *p* = 0.01, the generated network (WS) with *N* nodes and *m* * *N* edges has the small-world property. In numerical simulations, we set *N* = 1000 and *m* = 2, i.e., the average degree 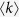 is equal to 4. BA networks can be constructed as follows: starting from *m*_0_ fully connected nodes, a new node with *m*(*m* ≤ *m*_0_) edges is added to the existing network at each time step according to the preferential attachment, i.e., the probability of being connected to the existing node *i* is proportional to its *k*_*i*_. We set *N* = 1000 and *m*_0_ = 3, *m* = 2, i.e., the average degree 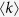 is about 4.

**Figure 2:**
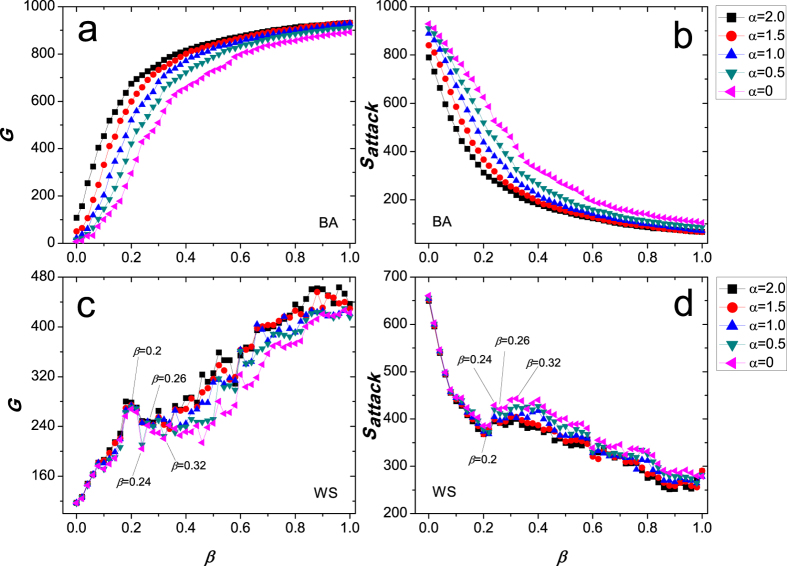
Cascading failures in BA scale-free networks and WS small-world networks.

In Fig. 2, we discuss cascading failures in BA scale-free networks and WS small-world networks, as triggered by the removal of the node with the highest load. The damage caused by a cascade is quantified by two measures, i.e., the largest connected component *G* and the average number *S*_*attack*_ of the failed nodes. After the removal of one node with the highest load, the redistribution of other nodes in turn may lead to the propagation of failures throughout the network. In Fig. 2(a,d), by gradually increasing the value of the capacity parameter *β*, We compare the effect of the parameter *α* on the robustness of BA networks and WS networks in five cases of *α* = 0, *α* = 0.5, *α* = 1.0, *α* = 1.5, and *α* = 2.0. When *α* = 0, the cascading model in Ref. 31 is a special case of our new model. Each curve is obtained by averaging over experiments on 20 independent networks. By two measures of *G* and *S*_*attack*_, we can clearly see that, as the value *α* increases, the robustness of BA networks and WS networks is stronger. In addition, we observe an interesting phenomenon, i.e., the ability paradox in the cascading model. According to the definition of the cascading model, the larger the value of the parameter *β*, the stronger the capacity of each node in a network. Generally, we intuitively think that, the stronger the capacity of each node in a network, the stronger the robustness of the whole network against cascading failures. While in WS networks (Fig. 2(c,d)), the numerical results are not what we expected. There exist some unusual data points in every curve of *α* = 0, 0.5, 1.0, 1.5, 2.0, for example *G* and *S*_*attack*_ in the cases of *β* = 0.24 (from *β* = 0.2 to *β* = 0.24) and *β* = 0.32 (from *β* = 0.2 to *β* = 0.24) when *α* = 0. From most previous cascading models, it is difficult to understand this strange phenomenon. In Fig. 2, we observe that this ability paradox only exists in WS networks. In other words, this interesting phenomenon is not universal in all networks. Using our cascading model, we further investigate cascading failures in real networks, i.e., a US airport network and two power grids.

**Figure 3:**
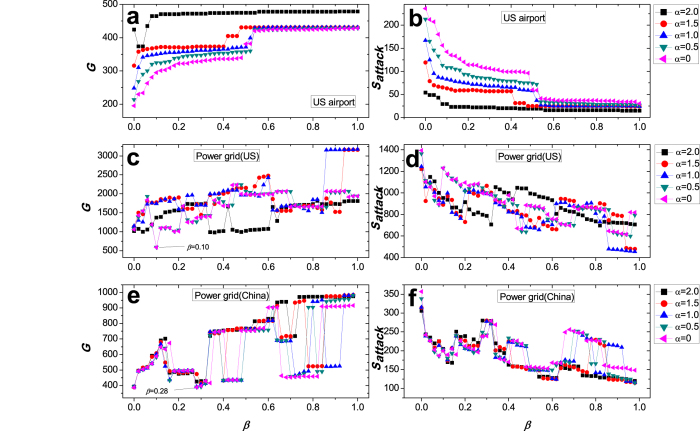
Cascading failures in a US airport network and two power grids.

In Fig. 3(a–f), we investigate cascading failures in a US airport network and two power grids (the power grid of five provinces in China southern with 1658 nodes and 4116 edges^68^ and the power grid in the western United States with 4941 nodes and 6594 edges^67^), as triggered by the removal of the node with the highest load. The legends and other parameters are the same as Fig. 2. In the US airport network (Fig. 3(a)), only when *α* = 2.0, we can observe the phenomenon of the ability paradox from 0 to 0.02 (values of *β*). While in two power grids (Fig. 3(c–f)), all curves show the obvious and wide fluctuation, which shows that sometimes investing more resources to protect a network is often played an opposite effect. For example, according to the largest connected component *G* in the US power grid (Fig. 3(c)), the network robustness of *β* = 0 is significantly stronger than that of *β* = 0.10 in the case of *α* = 0, which means that investing more protected resources makes the network more fragile. Similarly, in the power grid of five provinces in China southern (Fig. 3(e)), we can see that the values of *G* in two cases of *β* = 0 and *β* = 0.28 are almost same. Next, a natural question arises: what causes this counterintuitive phenomenon? We carefully analyze the cascading dynamical mechanisms, and speculate that some types of the local topology in a network may be the culprit of the ability paradox. We further try to explain this counterintuitive phenomenon by the local topology in a network.

**Figure 4:**
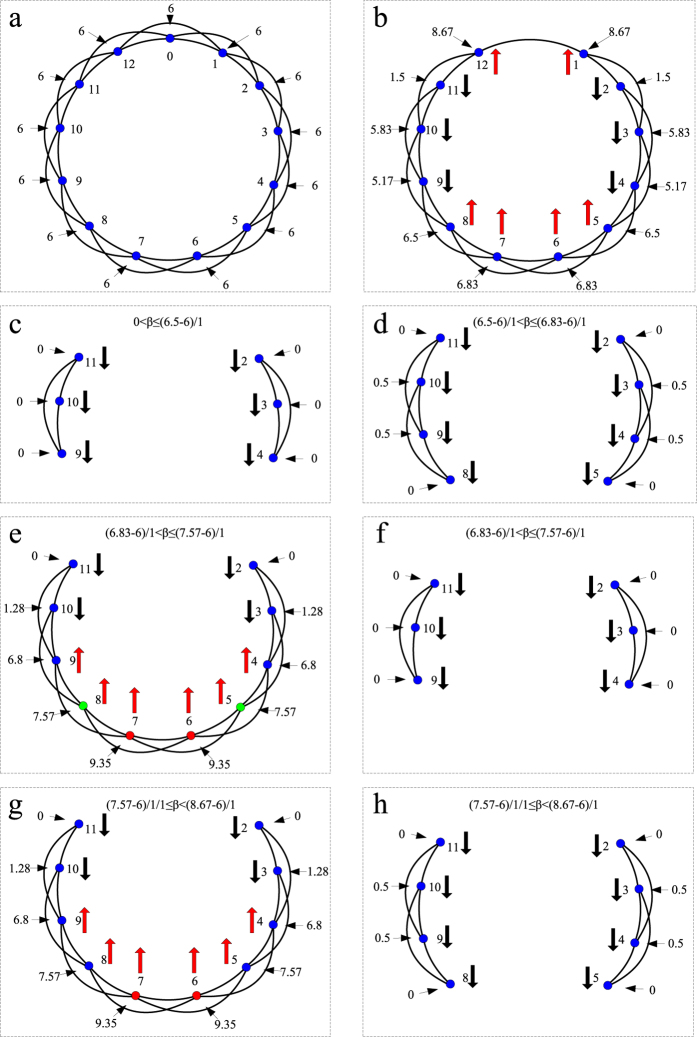
Analysis of the ability paradox in a coupled-ring network with 13 nodes and 26 edges.

**Figure 5:**
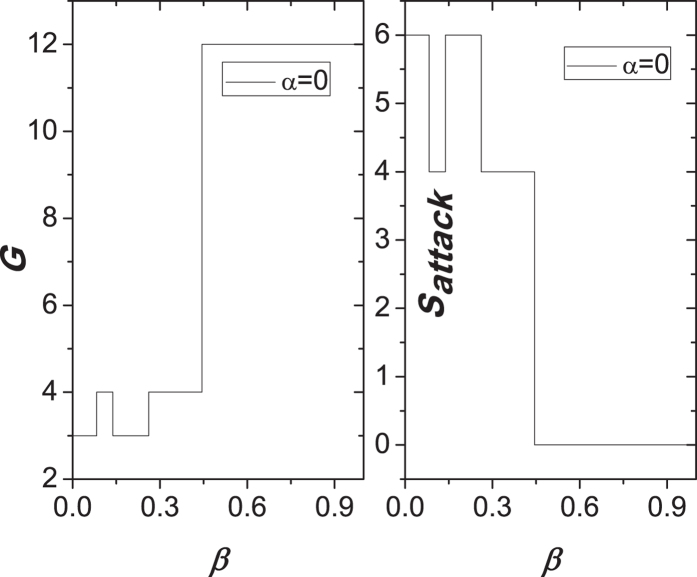
Correlation between two measures (*G* and *S*_*attack*_) and the parameter *β* after removing a node in Fig. 4.

Firstly, using a coupled-ring network with 13 nodes and 26 edges, we analyze the interesting phenomenon of the ability paradox by the load change after removing one node with the highest load. In every sub-figures in Fig. 4, the numbers inside and outside the coupled-ring network represent the labels of nodes and the initial load on each node in the case of *α* = 0 in our model. (*a*) In the initial state, the initial load on each node is 6. (*b*) After node 0 is removed from the network, we recalculate the load on each node and label the fluctuation of the load on each node by arrows, of which red arrow up represents that the load on a node increases, and black arrow down represent that the load on a node decreases, compared with the initial state in *a* sub-figure. In *c*, *d*, *e*, *f*, *g*, and *h* sub-figures, as values of the parameter *β* increase, we describe the cascading dynamical process of our model. (*c*) When 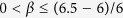, by analyzing *a* sub-figure, we can find that the loads on nodes 1, 5, 6, 7, 8, and 12 exceed their capacities, thus these nodes are removed from the network. After those nodes fail, because that the loads on the remaining nodes 2, 3, 4, 9, 10, and 11 do not exceed their capacities, no cascading failures occur and the system including nodes 2, 3, 4, 9, 10, and 11 maintains its normal and efficient functioning. Thus, in *c* sub-figure, the largest connected component *G* and the number *S*_*attack*_ of the failed nodes are 3 and 6, respectively. (*d*) By the similar analysis, when 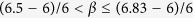, we can obtain that the values of *G* and *S*_*attack*_ are 4 and 4, respectively. (*e*) when 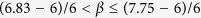, by analyzing *a* sub-figure, we find that, nodes 1 and 12 fail in the first stage of the cascading propagation. Then we recalculate the load on each remaining node (see *e*) and find that the loads on nodes 5, 6, 7, and 8 exceed their capacities. (*f*) Therefore, in the second stage of the cascading propagation, nodes 5, 6, 7, and 8 are removed from the network, and because that the loads on the remaining nodes 2, 3, 4, 9, 10, and 11 do not exceed their capacities, no cascading failures occur and the system including nodes 2, 3, 4, 9, 10, and 11 maintains its normal and efficient functioning. Finally, we get that the values of *G* and *S*_*attack*_ are 3 and 6, respectively. As the value of *β* increases from 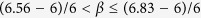 to 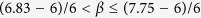, interestingly we observe that, the robustness of the network is weaken. By analyzing *e* sub-figure again, we find that, after the first stage of cascading failures occurs, it is because of the survivals of nodes 6 and 7 lead to the failures of nodes 5 and 8 (labeled by green circles), which maintain their normal and efficient functioning in *d* sub-figure. (*g*) By the similar analysis above, when 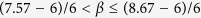, we give the states of every remaining nodes after the first stage of the cascading propagation. (*h*) After the second stage of the cascading propagation in *g* sub-figure, we can obtain that the values of *G* and *S*_*attack*_ are 4 and 4, respectively. When 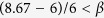, the removal of node 0 does not trigger the failure of other nodes. Thus, the network eventually stabilize at the state of *b* sub-figure. Therefore, When 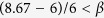, we can get that the values of *G* and *S*_*attack*_ are 12 and 0, respectively. In Fig. 5, we graphically give the correlation between two measures (*G* and *S*_*attack*_) and the parameter *β* after removing a node in Fig. 4. Similar with some curves in Fig. 3, we also see that two curves show the obvious and wide fluctuation, which means that there exist the phenomena of the ability paradox in Fig. 4. By the analysis of Fig. 4, we speculate that this ability paradox is mainly originated from that the resurrections of some nodes lead to a sharp increase of the load on other nodes, and further trigger the failures of these nodes due to not handle the extra load.

**Figure 6:**
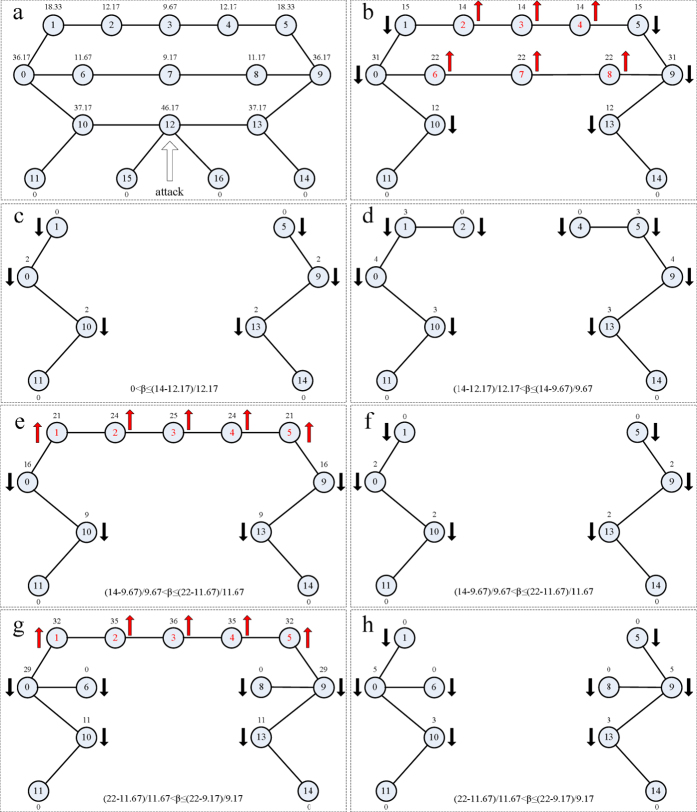
Analysis of the ability paradox in a network with the typical topology structure.

**Figure 7:**
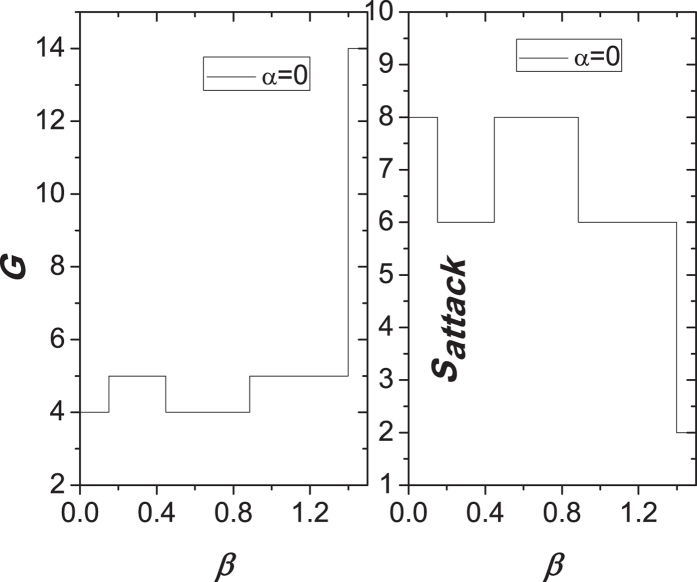
Correlation between two measures (*G* and *S*_*attack*_) and the parameter *β* after removing the node with the highest load in Fig. 6.

A fundamental question is, what type of the local network structure can lead to the phenomenon of the ability paradox? By analysis of the Figs 3 and 4, we speculate that the ring structure after removing a node may trigger this unusual phenomenon. To this end, we construct a simple network with the ring structure after removing a node. (*a*) We first calculate the initial load on every node in the case of *α* = 0 and remove the node (node 12) with the highest load (46.17). (*b*) We recalculate the load on every remaining node, of which red arrow up represents that the load on a node increases, and black arrow down represent that the load on a node decreases, compared with the initial state in *a* sub-figure. (*c*) When 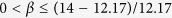, nodes 2, 3, 4, 6, 7, 8, 15, and 16 fail. We obtain that *G* and *S*_*attack*_ are 4 and 8, respectively. (*d*) When 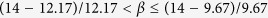, nodes 2 and 4 resurrect. Therefore, we get that the values of *G* and *S*_*attack*_ are 5 and 6, respectively. (*e*) When 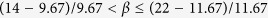, the resurrection of node 3 leads to a sharp increase of the load on nodes 2, 3, and 4. (*f*) Because nodes 2, 3, and 4 can not handle the extra load on them, they fail. Thus, we can get that the values of *G* and *S*_*attack*_ are 4 and 8, respectively. (*g*) When 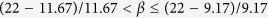, nodes 6 and 8 resurrect. And the load on nodes 1, 2, 3, 4, and 5 sharply increases. (*h*) When cascading failures stop, we get that the values of *G* and *S*_*attack*_ are 5 and 6, respectively. When 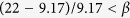, the removal of node 12 does not trigger the failure of other nodes. Thus, the network eventually stabilize at the state of *b* sub-figure. Therefore, we can get that the values of *G* and *S*_*attack*_ are 14 and 2, respectively. Similarly, in Fig. 7, we graphically give the correlation between two measures (*G* and *S*_*attack*_) and the parameter *β* after removing a node in Fig. 6. We clearly see that two curves show the obvious and wide fluctuation. By the analysis of Figs 3,4 and 7, we think that the phenomenon of the ability paradox is common in the network with the ring structure after removing a node, because in this type of network, some resurrection nodes due to the characteristics of the shortest paths easily lead to a sharp increase of the load on other nodes, and further trigger the failures of more nodes, while these nodes maintain their normal and efficient functioning in the case of their lower capacities.

**Figure 8:**
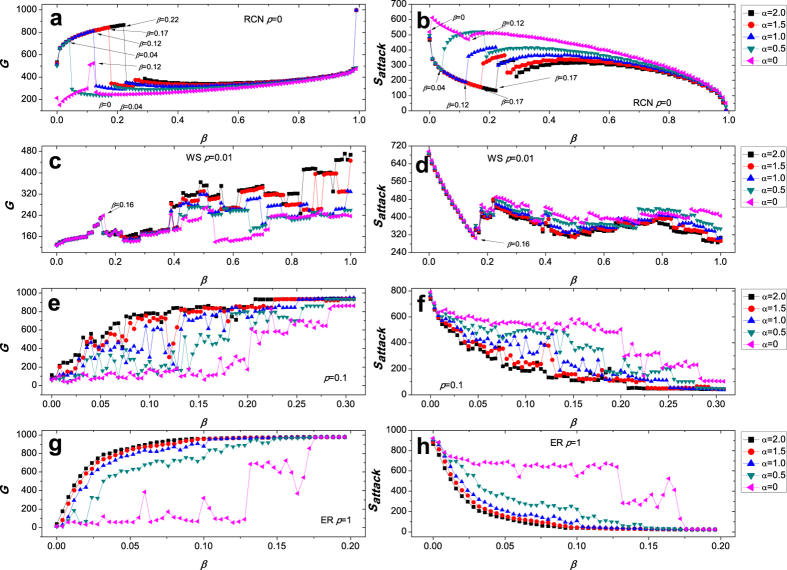
Cascading failures in four types of networks, initially constructed by a ring-coupled network (RCN) with 1000 nodes.

Starting from the coupled-ring network, we further analyze the universality of this phenomenon. In Fig. 8(a–h), we explore cascading failures in four types of networks, as triggered by the removal of the node with the highest load. We construct a ring-coupled network (RCN) with 1000 nodes (Fig. 8(a,b)), of which each node is connected with its 4 neighbors. For each link, there is then a rewiring probability *p*. Each curve corresponds to a realization of the network. Firstly, in Fig. 8(a,b), we investigate the effects of the parameters *α* and *β* on the robustness of a ring-coupled network (*p* = 0). From the values of *G* and *S*_*attack*_, we can observe that, the bigger the value of *α*, the stronger the robustness of RCN. with the increase of the value of *β*, we clearly see the ability paradox of cascading model at many points, for example, when *β* = 0, *β* = 0.04, *β* = 0.12, *β* = 0.17, and *β* = 0.22 correspond to *α* = 0, *α* = 0.5, *α* = 1.0, *α* = 1.5, and *α* = 2.0, respectively. In Fig. 8(c,d), different from Fig. 2(c,d), we perform an experiment and obviously observe the interesting phenomenon of the ability paradox of cascading model, e.g., the abnormal curves at the data point *β* = 0.16 for five different values of *α*. Similarly, in Fig. 8(e,f), as the value of *β* increases, five curves fluctuate more frequently. While in Fig. 8(g,h), only when *α* = 0, *α* = 0.5, and *α* = 1, there is the phenomenon of the fluctuation in three curves. This ability paradox of the cascading propagation might shed some new light on the analysis and control of cascading failures in real-world networks.

## Conclusion

In summary, we introduce a new method to assign the initial load on each node in a network and proposed a cascading model. By investigating the cascades of overload failures triggered by attacks on or failures of highly loaded nodes, we focus on two fundamental and practically important questions: in the same network, how the weight parameter on a node affects the robustness of a network against cascading failures and, under the disturbance of the global shortest paths, whether the enhanced capability on each node in a network must improve the robustness of the network against cascading failures. In some man-made networks and infrastructure networks, we investigate the cascading propagation induced by attacks on a single node with the highest load by two measures of the largest connected component and the number of the failed nodes. We have shown that the size of the cascade can be reduced by increasing the value of the weight parameter on the node in those networks. Specially, in two networks (BA scale-free networks and the US airport) induced by the network structure, the bigger the value of the weight parameter on a node, the stronger the robustness of these networks, however, in some networks (networks generated by a coupled-ring network and two power grids) induced by the network structure, we observe an interesting phenomenon about the ability paradox of our model, i.e., investing more protecting resources to protect the network may not be able to improve the robustness of the network against cascading failures. By carefully analyzing this phenomenon and the cascading dynamics of our model, we give an explanation that the topological structure of the ring with many nodes due to some resurrection nodes may lead to the invalid investment strategy. These results should be useful in furthering studies in the analysis and control of cascade failures in real-world networks.

## Additional Information

**How to cite this article**: Wang, J. *et al.* Ability paradox of cascading model based on betweenness. *Sci. Rep.* **5**, 13939; doi: 10.1038/srep13939 (2015).
